# Identification of Interpretable Clusters and Associated Signatures in Breast Cancer Single-Cell Data: A Topic Modeling Approach

**DOI:** 10.3390/cancers16071350

**Published:** 2024-03-29

**Authors:** Gabriele Malagoli, Filippo Valle, Emmanuel Barillot, Michele Caselle, Loredana Martignetti

**Affiliations:** 1 Institut Curie, Inserm U900, Mines ParisTech, PSL Research University, 75248 Paris, France; gabriele.malagoli@edu.unito.it (G.M.); emmanuel.barillot@curie.fr (E.B.); 2 Physics Department, University of Turin and INFN, 10125 Turin, Italy; filippo.valle@unito.it

**Keywords:** topic modeling, hierarchical stochastic block modeling, single-cell RNA-seq, long non-coding RNAs, breast cancer

## Abstract

**Simple Summary:**

Topic modeling, widely used in natural language processing, categorizes text documents into themes based on word frequency analysis. It has found success in various biological data analyses, including the accurate prediction of cancer subtypes and the simultaneous identification of genes, enhancers, and cell types from sparse single-cell data. Our study introduces a novel topic modeling approach for clustering single cells and detecting gene signatures in multi-omics single-cell datasets. Applied to study transcriptional heterogeneity in breast cancer cells resistant to chemotherapy and targeted therapy, it identifies protein-coding genes and long non-coding RNAs grouping cells into biologically similar clusters, effectively distinguishing between drug-sensitive and -resistant cancer types. Previous studies have interrogated long non-coding RNA (lncRNA) expression in single-cell data within breast cancer subtypes. Yet, the combined analysis of both lncRNA and mRNA expression in a cell type-specific manner remains to be explored. Compared to standard clustering methods, our approach offers a simultaneous optimal partitioning of genes and cells into topics and clusters, yielding easily interpretable results. Integrating mRNA and lncRNA data enhances cell classification accuracy.

**Abstract:**

Topic modeling is a popular technique in machine learning and natural language processing, where a corpus of text documents is classified into themes or topics using word frequency analysis. This approach has proven successful in various biological data analysis applications, such as predicting cancer subtypes with high accuracy and identifying genes, enhancers, and stable cell types simultaneously from sparse single-cell epigenomics data. The advantage of using a topic model is that it not only serves as a clustering algorithm, but it can also explain clustering results by providing word probability distributions over topics. Our study proposes a novel topic modeling approach for clustering single cells and detecting topics (gene signatures) in single-cell datasets that measure multiple omics simultaneously. We applied this approach to examine the transcriptional heterogeneity of luminal and triple-negative breast cancer cells using patient-derived xenograft models with acquired resistance to chemotherapy and targeted therapy. Through this approach, we identified protein-coding genes and long non-coding RNAs (lncRNAs) that group thousands of cells into biologically similar clusters, accurately distinguishing drug-sensitive and -resistant breast cancer types. In comparison to standard state-of-the-art clustering analyses, our approach offers an optimal partitioning of genes into topics and cells into clusters simultaneously, producing easily interpretable clustering outcomes. Additionally, we demonstrate that an integrative clustering approach, which combines the information from mRNAs and lncRNAs treated as disjoint omics layers, enhances the accuracy of cell classification.

## 1. Introduction

The recent development of single-cell technologies has enabled us to achieve a highly detailed view of the transcriptome in many biological studies. A fundamental step in the analysis of single-cell transcriptome data is the identification of cell populations, i.e., groups of cells that show “similar” expression patterns. 

To solve this task, many popular algorithms for unsupervised clustering have been implemented, which still present some computational challenges, such as estimating the optimal number of cell types [1] or the biological interpretation and annotation of the identified clusters [2].

A novel approach, based on topic modeling, has been recently introduced for simultaneously discovering clusters and interpretable gene signatures from bulk [3,4] and single-cell [5] transcriptomic datasets. Importantly, the algorithm used here for topic modeling, called hierarchical stochastic block modeling (hSBM) [6], is able to determine the optimal number of clusters (cell subpopulations) at different levels of resolution (i.e., the algorithm is able to reconstruct the hierarchical organization of cells into clusters) without a priori setting the number of expected clusters, and provides the topics (i.e., sets of genes) significantly associated with the identified clusters.

Here, we apply the hSBM algorithm to study the transcriptional heterogeneity of breast cancers and the role of long non-coding RNAs (lncRNAs) in contributing to this heterogeneity. LncRNAs are a large class of transcripts that are expressed in human cells without coding potential [7,8]. In healthy tissues, they have been shown to have lower expression, increased tissue specificity, and greater expression variability across individuals than protein-coding genes [9,10]. In cancer, lncRNAs are commonly dysregulated and their aberrant expression has been shown to be tumor type-specific [11,12]. However, the expression of lncRNAs at single-cell resolution has not yet been extensively studied. It is still unclear whether lncRNAs are highly expressed in subsets of cells within tissues, despite appearing lowly expressed in bulk populations. A single-molecule, single-cell RNA FISH survey of 61 lncRNAs in three human cell types showed that the low abundance of lncRNAs in bulk population measurements is not due to a small subpopulation of cells expressing lncRNAs at high levels, and overall lncRNAs are no different than mRNAs in their levels of cell-to-cell heterogeneity [13]. On the other hand, a larger scale study based on Smart-seq-total technology [14] has assayed a broad spectrum of mRNAs and lncRNAs from single cells and showed that the lncRNA content of cells significantly differs across cell types and dynamically changes throughout cellular processes such as cell cycle and cell differentiation. Another large-scale study, combining bulk tissue RNA-seq and scRNA-seq to deeply profile lncRNA expression during neocortical development, found that many lncRNAs are specific to distinct cell types and abundantly expressed in individual cells [15], thus supporting that the cell type-specific expression of lncRNAs contributes to the low levels of lncRNAs observed in tissues. Our work corroborates the latter kind of result.

Understanding breast cancer heterogeneity at the single-cell level is crucial in modern precision medicine [16,17]. Breast cancer is not a single disease; it encompasses a spectrum of molecular subtypes with diverse clinical behaviors and responses to treatment. Single-cell studies allow for a more granular understanding of the molecular landscape within individual tumors, enabling personalized treatment strategies tailored to each patient’s unique tumor characteristics. Moreover, single-cell analysis can identify specific cell populations within tumors, such as cancer stem cells or therapy-resistant cells, which may play critical roles in tumor progression, metastasis, and treatment resistance [18]. Targeting these specific cell populations could lead to more effective therapies and improved patient outcomes.

Previous studies [19,20] have documented strong correlations between lncRNA expression and breast cancer subtypes. Additionally, investigations have examined lncRNA expression in single-cell RNA-seq data within these subtypes [20]. However, the combined analysis of both lncRNA and mRNA expression in a cell type-specific manner has yet to be explored. Existing computational methods for analyzing single-cell RNA-seq (scRNA-seq) data do not consider the differences between protein-coding mRNAs and lncRNAs in terms of data dispersion and sparsity. Unsupervised clustering typically suffers from large fractions of observed zeros. In comparison to the analysis of mRNAs, the computational analysis of lncRNAs at the single-cell level is expected to be more challenging due to their high expression variability [2].

In our study, we used hSBM to investigate the transcriptional heterogeneity of luminal and triple-negative breast cancer cells using patient-derived xenograft models of acquired resistance to chemotherapy and targeted therapy [18]. We took advantage of this recent single-cell dataset that examined these two breast cancer subtypes in patient-derived xenograft (PDX) samples by generating therapy-sensitive and therapy-resistant PDX pairs. We therefore expected to be able to highlight the heterogeneity of cell subpopulations between subtypes but also within subtypes, thanks to a hierarchical clustering analysis.

First, we clustered the cells based on the expression of mRNAs and lncRNAs separately. We then used a multi-omics version of hSBM [4] to perform an integrative clustering that combines the information coming from the two RNA families treated as disjoint omics layers. We show that this strategy improves cell classification compared to clustering obtained by investigating mRNAs and lncRNAs separately, but also compared to clustering obtained on the expression measures of mRNAs and lncRNAs concatenated in a single matrix.

The main innovation we introduced is a reproducible and unsupervised procedure to investigate the gene content associated with cell clusters. For this purpose, we have developed a method to assign the most specific set of topics to each cluster and query them through gene set functional annotation databases (see Section 2). The topics have been interpreted in terms of functionally related gene sets of mRNAs and lncRNAs. This workflow allows for a deep investigation of the biological content of each cluster. The results show a clear enrichment of topics for pathways involved in the subtyping and progression of breast cancer and for sets of lncRNA that are interactors of important transcription factors involved in cancer. We have identified some lncRNAs associated with breast cancer cell subpopulations already well known in the literature, such as MALAT1 and NEAT1, and highlighted some others that may be clinically relevant.

## 2. Materials and Methods

### 2.1. scRNA-seq Dataset Pre-Processing

Single-cell expression count matrices were obtained from the NCBI GEO repository (GSE117309). This dataset includes scRNA-seq profiles from 3723 cells of patient-derived xenograft breast cancer models. Cells were collected from four cancer models: a luminal untreated drug-sensitive tumor, a luminal breast tumor with acquired resistance to Tamoxifen, a triple-negative tumor initially responsive to Capecitabine, and a triple-negative tumor with acquired resistance to Capecitabine.

Protein-coding mRNAs and lncRNAs raw expression matrices were obtained based on Ensemble grch37 gene annotations [21], after removing mitochondrial and ribosomal genes. Data were analyzed with Scanpy version 1.8.2 [22]. Library size normalization was applied and highly variable mRNAs and lncRNAs were selected according to the Scanpy procedure, selecting 1959 and 1136 features, respectively. We utilized two distinct thresholds for the parameter min_disp, considering the significant difference in the intrinsic coefficient of variation between the expression levels of the two gene families, as observed in our dataset (Supplementary Figure S1). 

### 2.2. hSBM Algorithm

We used the original hSBM clustering algorithm available at https://github.com/martingerlach/hSBM_Topicmodel/ (accessed on 1 March 2022) for topic modeling. The algorithm maximizes the probability that the model M describe the data A. Formally, it maximizes the quantity $P\left(A\right)=P\left(A|M\right)P\left(M\right)$. In our context, A is the gene expression matrix and the model M is composed of the levels of the hierarchy, each with its own partitions of genes and cells. hSBM minimizes the description length of the model, defined as $\Sigma =-lnP\left(M\right)lnP\left(M\;|\;A\right)$. The most important hyperparameter in the hSBM implementation of [6] is the number of initializations, i.e., the number of different starting points from which the algorithm begins to estimate the posterior probability $P\left(A\right)$. We decided to run hSBM with seven initializations for each experiment because we found that seven initializations best balanced the time cost and the ability to reach the minimum description length (see Supplementary Figure S2A). Multibranch SBM is the multipartite extension of hBSM: it works with a multipartite graph and finds partitions on each side of the graph [4]. We also ran multibranch SBM with seven initializations since it also shows an elbow at seven initializations (Supplementary Figure S2B).

### 2.3. Multibranch SBM Algorithm

The analytical framework for adding multiple layers of information to a hSBM inference problem was recently introduced for text analysis [23]. In the context of scRNA-seq data analysis, the algorithm was adapted to handle a tripartite network where nodes represent mRNAs, lncRNAs, and cells. mRNAs and lncRNAs were connected to cells according to their expression level, and therefore there was no edge between the mRNAs and lncRNAs nodes. The statistical inference procedure, as well as the definition of topics and probability distributions, leading to the tripartite network clustering is an extension of the hSBM algorithm. The n-partite SBM, introduced in [3], extends the hSBM algorithm described in [24]. Its implementation is available on gitHub at https://github.com/BioPhys-Turin/nsbm (accessed on 1 March 2022).

The inference procedure is performed using the functions implemented in the graph-tool library [24]. This algorithm infers the block structure on a given network, using a Markov Chain Monte Carlo (MCMC) that moves nodes (i.e., genes, lncRNAs, and cells) between blocks in order to minimize the so-called description length (DL) Σ [25]; this represents, in nat units, the number of bits that the model needs to describe the data. It can be written as the logarithm of the product of the likelihood times the prior $\Sigma =ln\left(P\left(A|\;M\right)P\left(M\right)\right)$ (A is the matrix of the data, and M represents all the model parameters) [25].

This description length Σ can be optimized [26] through a MCMC whose moves are accepted with the following probability:

(1) $$P\left(r\to s\;|\;t\right)=\frac{e_{ts}+\epsilon }{e_{t}+\epsilon B}.$$

The probability of moving a node from block (cluster or topic) *r* to a block s, given that a random neighbor of the considered node belongs to block *t*, is proportional to the ratio between the number of edges that connect group *t* and block *s* $e_{ts}$ and the number of edges that go to block *t* $e_{t}$, plus a term ϵB that is necessary to have a uniform probability if there are no connections between the blocks considered.

hSBM and multibranch SBM impose that nodes of a given type (mRNAs, lncRNAs, or cells) must not be mixed, so the blocks can contain only homogeneous nodes. This allows us to define clusters (blocks of cells), mRNA-Topics (blocks of mRNAs), and lncRNA-Topics (blocks of lncRNAs).

Once the model is run, moving all the nodes multiple times and accepting or refusing the moves with the aforementioned probability, it is possible to extract the P(mRNA | mRNA-Topic) and P(mRNA-Topic | cell) as well as P(lncRNA | lncRNA-Topic) and P(lncRNA-Topic | cell).

The P(mRNA | topic) is the ratio of the number of edges connected to the mRNA and the number of edges connected to the topic (the total number of edges connected to any gene in the topic). P(topic | cell) is the ratio of the total number of edges connected to the cell and the number of edges connecting the cell to the topic. The same definitions apply to P(lncRNA | lncRNA-Topic) and P(lncRNA-Topic | cell).

Finally, let us highlight that looking at the probability of accepting a move $P\left(r\to s\;|\;t\right)$, the advantage of using multibranch SBM on multi-omics problems is clear: when moving a cell from block r to block s, the algorithm samples a neighbor of the cell and it labels its group as *t*. At this point, the model only considers the links between the cell and the nodes in group t, so the normalisations of other branches are irrelevant. In other words, at each move, cells are clustered looking at a single omics and this allows us to naturally perform inference on multi-omics datasets in which each omic has a different normalization.

### 2.4. Normalized Mutual Information Score

To evaluate clustering performance, we computed the normalized mutual information (NMI), as defined in [27], between the partition found by the algorithm and the tumor of origin of the cells. NMI is a widely used measure to compare clustering methods [28]. However, adjustment for the so-called selection bias problem is needed; that is, NMI steadily increases with the number of clusters. To keep this effect into account, we adjusted the empirical NMI with the NMI* obtained with a null model that preserves the number of clusters and their sizes but reshuffles the labels of samples. Thus, NMI/NMI* represents how much the empirical score is higher than the score obtained with random populated clusters of the same size and number. This is particularly necessary when few original labels must be predicted: the lower the number of ground truth labels, the higher the chances of having a good prediction even with a random classifier. Two classifiers may have the same absolute NMI, but the one with a lower ratio NMI/NMI* resembles the prediction of a random model, meaning that the latter is less reliable.

### 2.5. Topic to Cluster Assignment

We developed a procedure to assign to each cluster its more suitable topics, exploiting the $P\left(topic\;|\;cell\right)$. 

First, we computed the $P_{c}\left(topic\;|\;cell\right)$ because it has been shown that is more informative than the $P\left(topic\;|\;cell\right)$ (11). This is defined as

(2) $$P_{c\left(cell\right)}=P\left(cell\right)-\frac{1}{N_{cells}}\sum_{c\;in\;allcells}P\left(topic\;|\;c\right)$$

Then, we calculated the $P_{c}\left(topic\;|\;cluster\right)$ as the mean of $P_{c}\left(topic\;|\;cell\right)$ over the cells inside the cluster

(3) $$P_{c\left(cluster\right)}=\frac{1}{dim\left(cluster\right)}\sum_{cell\;in\;cluster}P_{c\left(cell\right)}$$

Finally, we assigned to each cluster all the topics that satisfied the condition

(4) $$P_{c}\left(cluster\right)>\mu +n*\sigma$$

where μ and σ are the mean and the standard deviation of the $P_{c}\left(topic\;|\;cluster\right)$ across all the topics for the cluster under examination.

The core of the procedure concerns the choice of n: to make it unsupervised and reproducible, we started from n=3, and then we lowered n by a step of 0.05 until two conditions were fulfilled; first, we required that at least one topic is assigned to each cluster, and second, that there were no clusters with the same sets of topics, but subsets were allowed. It is worth noting that following this method, it is possible to assign to any kind of partitions the most important topics for each subset: for example, one could group cells by subtype and, by computing the $P_{c}\left(topic\;|\;subtype\right)$, could identify the most important topics for each tumor subtype. 

### 2.6. Functional Enrichment of Topics

The gene content of topics was tested through hypergeometric statistics for functional enrichment using annotations from the MsigDB Molecular Signature Database version 7.5.1 (MsigDB) [29] and the lncSEA database [30]. We tested all topics assigned to the clusters, excluding those not associated with any of the cell partitions. After correcting hypergeometric test *p*-values for multiple testing using Benjamini–Hochberg [31], we associated each topic with the most enriched functional gene set. When a gene set was associated with multiple topics, the one with the lowest false discovery rate was retained.

We observed a strong bias in favor of large-size gene sets as an outcome of the hypergeometric test when using the lncSEA database: most of the genes in the topics corresponded to the collection called “Accessible Chromatin”, more precisely, to gene sets consisting of more than 12,000 genes.

To investigate the impact of these large-size gene sets on our results, we studied the structure of the MsigDB and lncSEA databases. MSigDB contains 32,880 gene sets divided in 9 collections including a total of 40,755 genes. Collections correspond to a set of genes constructed by the same method or exploring similar biological information. For example, the collection “Hallmarks” (H) of MsigDB consists of “coherently expressed signatures derived by aggregating multiple gene sets to represent well-defined biological states or processes’’ [30]. LncSEA contains 41,365 gene sets divided into 18 collections and 58,478 genes. We computed the ratio between each set size and the total database size (the total number of genes in the database). The distributions of the ratios calculated for the two databases have a similar shape, but the maximum value of the ratio for MsigDB is 0.05 while for lncSEA it is 0.25 (Supplementary Figure S3A,B). This suggests that the lncSEA database comprises numerous gene sets with a large number of elements, ranging from 10,000 to 14,000, that may have questionable biological significance. To filter out extremely long gene sets, we calculated the ratio between the size of each gene set and the total number of genes within the collection which the gene set belongs to. All gene sets whose ratio was greater than 0.15 were filtered out. 

Nevertheless, this filtration method fails to tackle the problem of redundant gene content present within sets. To eliminate redundant sets, one possible solution is to calculate the Jaccard distance between each pair of gene sets and remove those that are more similar than a specific threshold. For a collection with n gene sets, this requires computing n(n + 1) operations, as each set’s intersection and union must be calculated with all others, resulting in 2 × n(n + 1)/2 operations. However, collections such as C11 and C13 in the lncSEA database contain around 14,000–18,000 genes, which makes this operation incredibly time-consuming and computationally demanding. When dealing with a total of approximately 70,000 gene sets between the two databases, this method becomes almost impractical. Nevertheless, we can demonstrate that, for a subset of classes, the Jaccard distance and gene set length are linked by a decreasing monotonic relationship. In other words, the longer a list, the more similar it is to other lists in the same class. Consequently, implementing the intersection-with-universe filter is a quick and accurate approximation of the Jaccard filter (Supplementary Figure S3C–F).

## 3. Results

### 3.1. Clustering of Drug-Sensitive and Resistant Breast Cancer Cells Using hSBM

To explore intra-tumor transcriptional heterogeneity in breast cancer, we used a dataset of scRNA-seq profiles of patient-derived xenograft (PDX) models of luminal and triple-negative breast cancer with acquired drug resistance [18]. After scRNA-seq data pre-processing (see Materials and Methods), we obtained two expression matrices containing, respectively, 1959 mRNAs and 1136 lncRNAs profiled in 3723 cells from drug-sensitive and -resistant tumors. We translated each expression matrix into a weighted bipartite network where nodes represent genes (mRNAs or lncRNAs) and cells. Genes are linked to the cells with weighted edges based on their level of expression. We applied the hSBM algorithm to calculate the best partitions of features and cells. The output of hSBM is a hierarchical and probabilistic organization of the network in blocks of connected genes and cells. In hSBM, the different levels correspond to different degrees of aggregation of nodes in the network into blocks or communities. These levels represent a hierarchy of community structures that can be identified within the network.

For each level of hierarchy, we obtained three matrices of probability membership, corresponding to the probability of cells to be associated with a cluster (which is a delta function since we set the model to output “hard” clusters), the probability of association of genes to topics, and the probability of association of a topic to a cell. In our context, the output of hSBM informed us about the best partitioning of cells into clusters with similar transcriptional profiles and about which genes were most strongly associated with these clusters.

**Figure 1:**
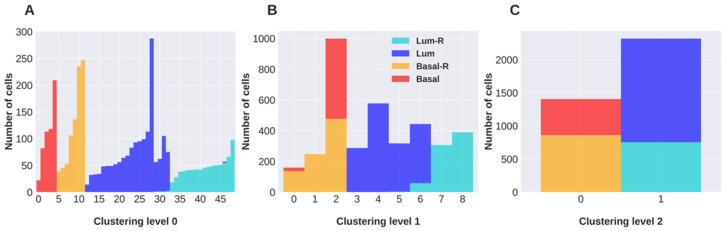
Clustering of cells obtained with hSBM applied to the mRNAs expression dataset. For each hierarchical level of clustering (**A**–**C**), columns report the size of the identified clusters and the tumor of origin of their component cells. Each color corresponds to a breast cancer subtype and the suffix -R indicates that the cancer has been treated to develop chemoresistance.

The partition of the network based on mRNA expression shows three hierarchical levels of cell classification (Figure 1A–C). In the hierarchical level 2 (Figure 1C), the cells are classified into two clusters containing, respectively, the luminal and triple-negative tumor cells, regardless of their resistant or drug-sensitive phenotype. Level 1 of clustering resolution (Figure 1B) separates cells into nine clusters, three of which contain a mixture of sensitive and resistant cells, suggesting that heterogeneous populations of sensitive and resistant cells emerged. The largest of these mixed clusters includes 999 triple-negative sensitive and resistant cells, while none of the mixed clusters contain both basal and luminal cells. Level 0 of classification (Figure 1A) separates cells into 49 clusters, all containing cells from specific tumor types.

**Figure 2:**
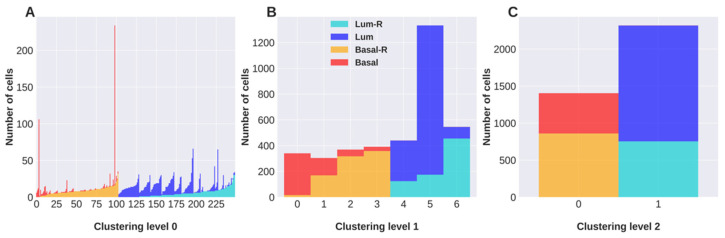
Clustering of cells obtained with hSBM applied to the lncRNA expression dataset. For each hierarchical level of clustering (**A**–**C**), columns report the size of the identified clusters and the tumor of origin of their component cells.

Also, the partition of the lncRNA-based network exhibits three hierarchical levels of cell classification (Figure 2A–C). Similar to the clustering of the mRNA network, level 2 (Figure 2C) classifies cells in two clusters with luminal and basal tumor cells. However, the hierarchical levels 1 and 0 show some differences compared to the classification based on mRNAs. Level 1 (Figure 2B) separates cells into seven clusters, all containing a mixture of resistant and sensitive cells. Level 0 of classification (Figure 2A) separates cells into 249 clusters, many of which include both resistant and sensitive cells.

To evaluate the performance of the hSBM clustering algorithm, we calculated a score based on the normalized mutual information (NMI) [25] between the partition found by the algorithm and the tumor of origin of the cells. NMI measures the agreement between two sets of discrete labels, one of which is considered the ground truth, the breast cancer subtype in this work. NMI* measures the quality of the prediction of a random classifier. The higher the ratio NMI/NMI*, the better the performances are and the further the classifier is from a random one (see Materials and Methods). The NMI/NMI* scores obtained for the mRNA-based and lncRNA-based clustering are reported in Table 1. The NMI/NMI* score decreases as clustering resolution increases from level 2 to level 0, as cells from the same tumor type are separated into different clusters. At level 2 and level 1 resolution, NMI/NMI* scores are comparable for clustering obtained with mRNAs and that obtained with lncRNAs, whereas at level 0, NMI/NMI* is an order of magnitude lower for clustering obtained with lncRNAs. The low NMI/NMI* obtained for level 0 of clustering with lncRNAs shows that their expression is not very informative of the tumor type at this clustering hierarchical level. Instead, the classification of cells obtained at level 1 provides information strongly correlated with tumor types. Remarkably, the mRNA-based clustering divides cells differently than the lncRNA-based clustering, suggesting that the expression of these two RNA families carries complementary information about cell heterogeneity. 

### 3.2. Cells Are Better Classified by Analyzing the Expression of mRNAs and lncRNAs as Separate Omics Layers

We then simultaneously used mRNA and lncRNA expression data to perform integrative clustering. These data are generated on the same platform in a single experiment, and the standard approach for integrative analysis is to concatenate the expression measurements into a single matrix on which to apply the clustering algorithm. A more sophisticated approach would consider the inherent difference between mRNA and lncRNA expression and treat the two RNA families as disjoint omics layers. Indeed, we verified that in this dataset, lncRNAs show statistically higher coefficients of variance than mRNAs (Supplementary Figure S1). 

To integrate the information coming from mRNAs and lncRNAs, we designed two different experiments. In the first one, we concatenated the mRNA and lncRNA expression measurements into a single matrix of 3095 features and applied the hSBM algorithm on it. In the second experiment, the mRNA and lncRNA matrices were treated as disjoint omics layers and a multi-omics version of hSBM was applied, that is called multibranch SBM [3] and is similar to the multilayer SBM algorithm used for text document analysis [4]. This extends the network-based topic modeling algorithm to a weighted tripartite network where nodes represent mRNAs, lncRNAs, and cells. Similarly to hSBM, mRNAs and lncRNAs are linked to the cells according to their expression level, while no mRNA-lncRNA edges are present, and the best partition of the tripartite network is computed. 

**Figure 3:**
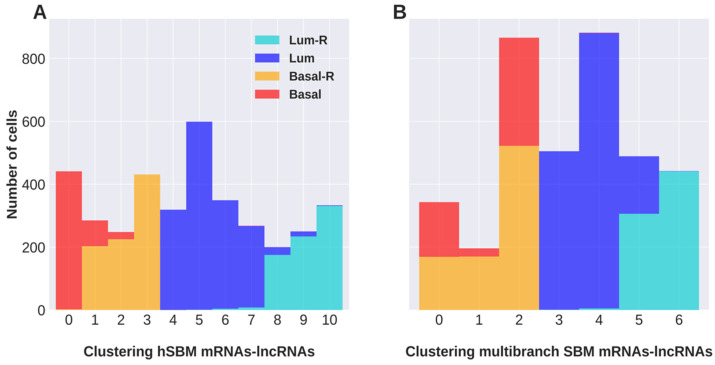
Clustering of cells at level 1 obtained with hSBM applied to the concatenated mRNA and lncRNA expression matrices (**A**) and with multibranch SBM applied to the mRNA and lncRNA matrices used as disjoint omics layers (**B**). Columns report the size of the identified clusters and the tumor of origin of their component cells.

The two experiments produced very different results in terms of cell partition (Figure 3). Considering level 1, based on hSBM applied to the concatenated expression matrices, we obtained eleven clusters almost exclusively composed of cells specific to one specific tumor type (Figure 3A). The multibranch SBM algorithm separated the cells into seven clusters, three of which contained a mixture of resistant and sensitive cells (Figure 3B). 

**Figure 4:**
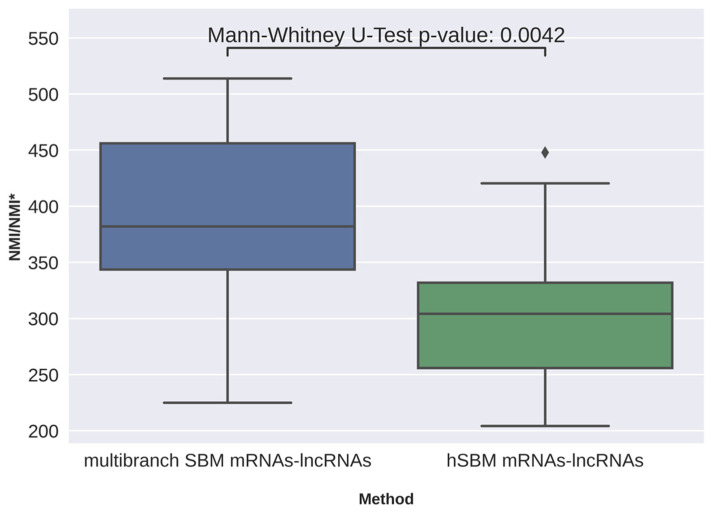
Boxplot showing the performances obtained with multibranch SBM and with hSBM applied to the concatenated matrices, both at resolution level 1.

The NMI/NMI* score of clustering level 1 (hSBM-mRNAs-lncRNAs) obtained for the hSBM applied to the concatenated matrices was lower than the one obtained for mRNAs and lncRNAs analyzed as disjoint omics layers (multibranch SBM mRNAs-lncRNAs) (Table 1). In the clustering obtained with the multibranch SBM algorithm, the NMI/NMI* score clearly improves compared to all previous experiments (Table 1), showing that mRNA and lncRNA matrices treated as disjoint omics layers provide more relevant information about tumor heterogeneity. The performances of the multipartite approach are not the best in level 0 and level 2 of the hierarchy. However, from all four experiments, these partitions show either a very rough clustering of the cells (level 2 always shows two clusters, the basal-like and luminal-like cells) or an extremely fine clustering with hundreds of groups, each one containing very few cells, the biological meaning of which is questionable. The difference between the tripartite experiment and the others was not very large, but we checked whether the tripartite approach leads to systematically better results than the bipartite approach. We ran each experiment (hSBM-mRNAs-lncRNAs and multibranch SBM-mRNAs-lncRNAs) multiple times, measuring the NMI/NMI* score. Each experiment was run 20 times and we excluded the runs with performances out of the 0.05 and 0.95 quantile in order to remove outcomes with highly unlikely scores. The results of the multiple runs show that multibranch SBM is statistically significantly better than the bipartite approach (Figure 4). Despite a slight increase in the NMI/NMI* fold change, the improvement in the classification measured with the NMI/NMI* score on multiple runs is systematically slightly better for the multibranch algorithm.

### 3.3. Functional Enrichment Analysis of Topics

We wanted to assess whether the gene content associated with the breast cancer cell clusters according to multibranch SBM was enriched for specific biological functions. One of the pitfalls of enrichment analysis is the selection of input genes to be tested for enrichment [32]. The common practice in transcriptome analysis is to define a threshold to apply to the *p*-values to select differentially expressed genes. The cut-off is arbitrary and it has been shown that the effect of threshold choice impacts biological conclusions reached during enrichment analysis [33].

The hSBM and multibranch SBM algorithms provide as output topics that can be used as input lists for the enrichment analysis. Gene lists associated with topics are defined during the simultaneous optimal assignment of cells to clusters and genes to topics, without any dependency on an arbitrary threshold.

**Figure 5:**
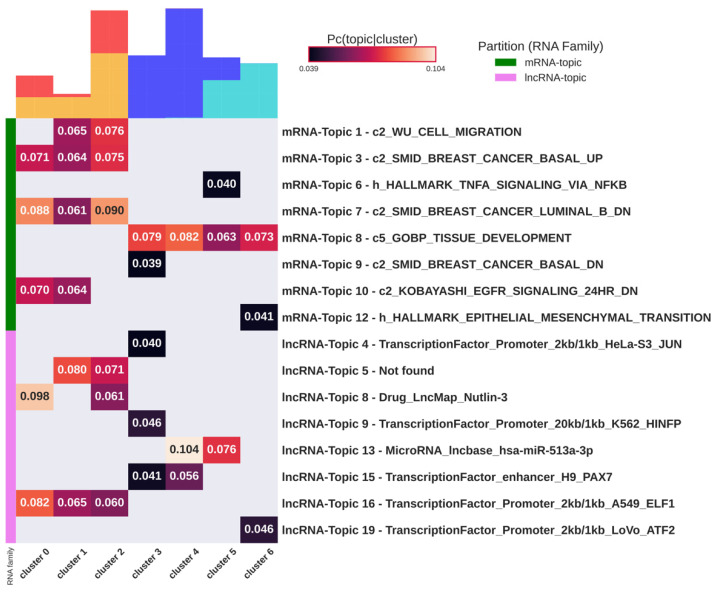
Functional enrichment analysis of topics. The heatmap reports the probability of association of topics to clusters (when statistically significant) and the most enriched functional gene. Green and pink lines highlight mRNA-Topics and lncRNA-Topics, respectively. On top of each column, the composition of the cluster is shown in terms of tumor subtype color coded as in Figure 1 (red = “Basal”; yellow = “Basal-R”; dark blue = “Luminal”; and light-blue = “Luminal-R”). All mRNA-Topics are associated with biological processes related to cancer and to gene signatures specific to breast cancer subtypes. The lncRNA-Topics are mainly associated with gene sets that represent interactors of transcription factors that play a role in cancer and in cell differentiation.

We tested through hypergeometric statistics the enrichment of topics for the gene functional annotations of the MsigDB Molecular Signature Database 7.5.1 (MsigDB) [29] and lncSEA database [30] for the mRNA-Topics and the lncRNA-Topics, respectively. We observed that the output of enrichment analysis is biased towards very long lists, and therefore we decided to filter out uninformative gene sets before the test (see Materials and Methods). One of the two main innovations that we introduced is an unsupervised and reproducible procedure to select the most specific topics to each cluster, based on the probability matrix of association of a given topic to a given cell (see Materials and Methods). The results of the topic enrichment analysis are provided in Figure 5. 

The results show a clear enrichment of all but one topic to the pathways involved in the subtyping and progression of breast cancer and to sets of lncRNA that are interactors of transcription factors involved in cancer. Notably, we found topics specifically associated with luminal or basal cell clusters, with no topics associated with both luminal and basal types. As an example, mRNA-Topic 3 shows the keyword SMID_BREAST_CANCER_BASAL_UP [34] and is enriched only in basal-related clusters. The same happens for mRNA-Topic 7 which, being also composed of basal cells, shows a keyword related to luminal downregulation. All mRNA-Topics and lncRNA-Topics are associated with clusters of a specific tumor type and can be used in an unsupervised framework to identify the tumor type.

mRNA-Topic 1 is enriched in genes involved in cell migration and specifically associated with a subpopulation of cells from basal tumors, supporting a higher migratory capacity of basal cells compared to luminal ones [35]. Gene content in mRNA-Topic 6 is enriched in HALLMARK_TNFA_SIGNALING_VIA_NFKB and specifically associated with cell cluster_5 containing a mixture of resistant and sensitive luminal cells. This result suggests the NF-kB regulation by TNFα as a transcriptomic hallmark common to a subset of resistant and sensitive luminal cells. Cell cluster_5 is also significantly associated with lncRNA-Topic 13, which includes MALAT1 and NEAT1 as top ranked genes, two lncRNAs extensively studied in breast cancer progression [36,37]. Another important result shows the association of cluster_6, composed only of therapy-resistant luminal cells, to mRNA-Topic 12 (HALLMARK_EPITHELIAL_MESENCHYMAL_TRANSITION) and lncRNA-Topic 19 (interactors of ATF2). The transcription factor ATF2 has been shown to induce therapy resistance to melanoma [38]. Finally, lncRNA-Topic 8, associated with two of the three basal clusters, consists of lncRNAs whose expression is correlated with the effect of Nutlin-3, a well-known compound used in studies for cancer treatment [39]. In Table 2, we summarized some functional lncRNAs known in the literature that are associated with the identified topics.

### 3.4. Topic Modeling versus Clustering Approach

In order to compare our method with a standard clustering approach, we decided to compare the topic modeling with the clustering algorithm available in Seurat [50]. Seurat calculates cell clustering based on a single omic or with integrated omics with a weighted-nearest neighbor (WNN) approach [50]. We applied Seurat for clustering cells based on mRNA expression and lncRNA expression alone and then for integrated clustering (Seurat-WNN) with mRNAs and lncRNAs used as disjoint omics layers. We compared results obtained from Seurat clustering on individual omics with Seurat integrated clustering. To do this, we computed the adjusted mutual information (AMI) [51], a score based on mutual information such as the NMI, but used when neither of the two sets of labels can be taken as ground truth. AMI varies between 0 and 1, 1 being the situation of maximal agreement between sets.

**Figure 6:**
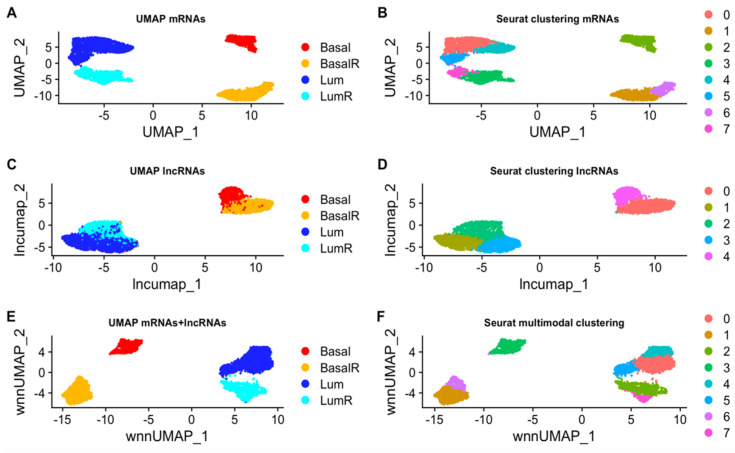
UMAPs representing the clustering obtained with Seurat using mRNA expression alone (**A**,**B**), lncRNA expression alone (**C**,**D**), and using both modalities (**E**,**F**). On the left is shown the tumor of origin of the cells in the UMAP and on the right the clusters obtained with Seurat. (**A**,**E**) are extremely similar, showing the irrelevant change when the lncRNA expression is taken into account.

In terms of ability to reconstruct the correct tumor type, Seurat WNN achieves better performance because the NMI/NMI* scores of the Seurat experiments are higher, as shown in Table 1. However, a deeper investigation showed that Seurat WNN does not fully exploit both omics, since the integrated clustering (Figure 6F) is very similar to the one obtained using mRNA expression alone (Figure 6B): both consist of eight clusters and each tumor type is separated into the same number of clusters. 

**Figure 7:**
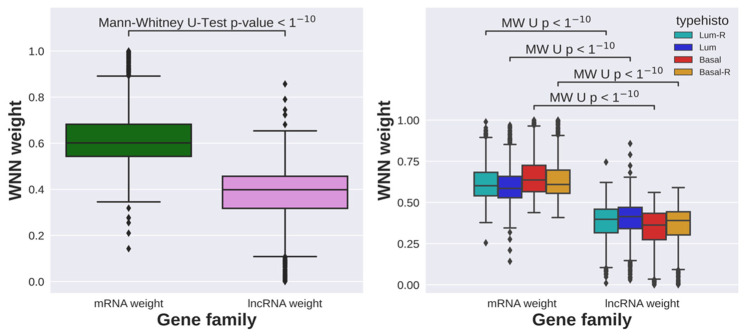
The boxplots plots show the weights assigned by Seurat WNN to the mRNAs and lncRNAs for all cells (**left panel**) and for cells grouped by tumor of origin (**right panel**). *p*-values are obtained with Mann–Whitney U-test.

The agreement (AMI) between Seurat WNN and Seurat applied to mRNA expression alone is 0.855 (Table 3). This shows that introducing lncRNAs does not significantly change the results because the agreement between the single matrix and the joint approach is very high. The AMI between the Seurat clustering applied to lncRNA expression alone (Figure 6D) and Seurat WNN clustering (Figure 6F) is 0.569 (Table 3), which indicates very divergent results. We might impute this divergence to the cell specific modality weights calculated by Seurat WNN, that learns the information content of each modality and determines its relative importance in downstream analyses [50]. The weights assigned to the mRNAs are significantly higher than those assigned to the lncRNAs (Figure 7), showing that mRNAs are predominantly used for the clustering.

On the other hand, multibranch SBM assigns equal importance to each modality without introducing weights and possibly biases. The agreement between the integrated clustering and the clustering on the individual omics hSBM-mRNAs and hSBM-lncRNAs are low, respectively, 0.492 and 0.493 (Table 3). This suggests that the proposed multi-branch SBM method can be used as a complementary method to analyze single-cell multi-omics data and extract information about cell subpopulations, because it equally weighs the modalities that are provided as input, without any bias towards one of them.

## 4. Discussion

The topic modeling procedure proposed here has proved to be a valid approach for the integrative and unsupervised clustering of protein-coding gene and lncRNA expression at the single-cell level. Multibranch SBM enables the classification of breast cancer cell populations, showing that mRNA and lncRNA datasets provide complementary information about the heterogeneity of the tumors under study, when they are treated as disjoint omics layers. 

The combination of several single-cell omics layers represents a new frontier for single-cell genomics and requires appropriate computational methods. Recently, several experimental techniques were capable of assaying multiple modalities simultaneously from the same single cells, including CITE-seq [52], 10X Genomics ATAC + RNA, SHARE-seq [53], SNARE-seq [54], and many others. The multibranch SBM algorithm can be applied in different contexts of integrative multimodal analysis to learn the information content of each modality in each cell and to define cellular states based on a combination of information derived from multiple modalities.

Differently from state-of-the-art clustering methods used in single-cell data analysis, such as the Leiden algorithm and graph-based algorithms [55,56], multibranch SBM not only provides an integrative clustering of cells based on both mRNA and lncRNA expression, but also outputs the probability distribution of the association of these features to clusters, that is, their relative importance in driving cell classification. Clusters of cells and sets of associated features (topics) are simultaneously identified with SBM algorithms. Topics can be used for functional enrichment analysis, avoiding the arbitrary cut-off on the *p*-values to define input lists. Another great advantage of the SBM algorithm is that it automatically detects the optimal number of clusters and hierarchically clusters both the features and cells. This prevents researchers from fixing the number of expected clusters, which is difficult in real datasets. In addition, we showed that clustering performed with multibranch SBM is not affected by the batch effect, a common problem affecting the analysis of large-scale single-cell transcriptomic datasets (Supplementary Figure S4). The comparison with Seurat WNN showed that we can better exploit the two omics layers whereas Seurat WNN relies more on the mRNA information. In our study of breast cancer PDX models, the highest hierarchical level separated the luminal and basal cells, while the second level showed a finer tumor heterogeneity. 

Compared to standard state-of-the-art clustering analysis, our method provides a simultaneous optimal partitioning of genes into topics and cells into clusters, allowing for interpretable clustering results. We proposed a new approach to investigate the heterogeneity of single cells, based on a reproducible and unsupervised method to associate with each cluster the set of topics that identifies the behavior of the cells within a cluster, removing the arbitrariness in the interpretation of the enrichment analysis. Identified topics associated with clusters are enriched for genes and pathways clearly involved in breast cancer subtyping, such as cell adhesion and migration, and in sets of lncRNAs that are interactors of transcription factors related to cancer. 

Overall, we identified some lncRNAs with a high probability of association with topics that are well known in the breast cancer literature and some other very interesting candidates for functional validation. In lncRNA-Topic 4, which includes a set of 19 lncRNAs, SIRLNT (SIRT1 regulating lncRNA tumor promoter) has the top high probability of association with the topic (P(SIRLNT|lncRNA-Topic 4) = 0.6). This lncRNA, also called lncRNA-PRLB (progression-associated lncRNA in breast cancer), is upregulated in human breast cancer tissues and breast cancer cell lines [40] Moreover, it has been described as a modulator of the SIRT1 gene both at the mRNA level and protein level. SIRT1 is a member of the sirtuin family of proteins, which have been extensively studied in multiple cancers [41], including breast cancer [41,42].

Also strongly associated with lncRNA-Topic 4 we found GATA3-AS1, a lncRNA recently proposed as a predictive biomarker of nonresponsive breast cancer patients to neoadjuvant chemotherapy [43]. GATA3-AS1 was described as a functional lncRNA and positive transcriptional regulator of *GATA3* in the TH2 lineage of lymphocytes [43].

lncRNA-Topic 8 is strongly associated with clusters 0 and 2, both of which are composed of triple-negative sensitive and resistant cells. LncRNA-Topic 8 presents among its top features some lncRNAs extensively studied in breast cancer, and particularly in triple-negative subtypes. The top ranked lncRNA in lncRNA-Topic 8 is LINP1, a regulator of DNA repair in triple-negative breast cancer [44], whose knockdown increases the sensitivity of breast cancer cells to radiotherapy [57]. A second relevant lncRNA belonging to lncRNA-Topic 8 is LINC00319, which has been shown to promote cancer stem cell-like properties [44].

LncRNA-Topic 13 is characterized by two lncRNAs associated with it with a very high probability that are well known in the literature, namely, MALAT1 (Metastasis Associated Lung Adenocarcinoma Transcript 1) and NEAT1. MALAT1 is one of the most studied non-coding RNAs in cancer, generally overexpressed in tumor progression and metastasis [36]. NEAT1 is also abnormally expressed in many cancers and associated with therapy resistance and poor clinical outcomes [45]. In our analysis, lncRNA-Topic 13 is significantly associated with cluster 5 which contains a mixed subpopulation of sensitive and therapy-resistant luminal cells, suggesting that a subset of sensitive cells share this lncRNA signature with resistant cells. 

The host gene for miR-205 (MIR205HG) is the most strongly associated with lncRNA-Topic 15, expressed in luminal sensitive cell cluster 1 and cluster 2. MIR205HG has independent functions as a lncRNA, regulating growth hormone and prolactin production in the pituitary gland [46]. Remarkably, MIR205HG was previously indicated as a predictor of anti-cancer drug sensitivity [46,47].

LncRNA-Topic 19 is specifically expressed in cluster 6 of drug-resistant luminal cells. The top ranked lncRNAs associated with lncRNA-Topic 19 are LINC00493, also known as SMIM26, and TP53TG1. LINC00493 and TP53TG1 are enriched and co-released in extracellular vesicles from colorectal cancer cells [48]. Previous studies have highlighted the ambivalent oncogenic or tumor suppressor activity of TP53TG1 in luminal breast cancer [49,58].

## 5. Conclusions

In conclusion, the topic modeling method introduced in this study has demonstrated its efficacy in integrating and clustering protein-coding genes and lncRNA expression in single cells in an unsupervised manner. Multibranch SBM allows for the classification of breast cancer cell populations, indicating that mRNA and lncRNA datasets offer complementary insights into tumor heterogeneity when treated as separate omics layers.

Using this method, we pinpointed protein-coding genes and lncRNAs that classify thousands of cells into biologically similar clusters, effectively discriminating between drug-sensitive and -resistant breast cancer subtypes. Compared to conventional clustering analyses, our approach achieves an optimal division of genes into topics and cells into clusters simultaneously, resulting in easily interpretable clustering results.

## Figures and Tables

**Table 1:** NMI/NMI* scores computed to assess the consistency between the cell clusters obtained from diverse experiments conducted with hSBM and Seurat algorithms, and the original tumor from which the cells were derived.

NMI/NMI*	Clustering Level 0	Clustering Level 1	Clustering Level 2
hSBM-mRNAs	66	325	1560
hSBM-lncRNAs	9	359	1642
hSBM-mRNAs-lncRNAs	53	294	1618
multibranch SBM mRNAs-lncRNAs	56	398	1603
Seurat-mRNAs	465		
Seurat-lncRNAs	614		
Seurat-WNN mRNAs-lncRNAs	451		

**Table 2:** LncRNAs associated with lncRNA-topics with a high probability have already been documented in the literature.

**lncRNA-Topic 4**		
**lncRNA_ID**	**Probability of association to topic**	**Ref.**
SIRLNT = CTA-392C11.1 = ENSG00000253802	P(SIRLNT|lncRNA-Topic 4) = 0.6	[40,41,42]
GATA3-AS1 = ENSG00000197308	P(GATA3-AS1|lncRNA-Topic 4) = 0.05	[43]
**lncRNA-Topic 8**		
**lncRNA_ID**	**Probability of association to topic**	**Ref.**
LINP1 = RP11-554I8.2 = ENSG00000223784	P(LINP1|lncRNA-Topic 8) = 0.12	[44]
LINC00319 = ENSG00000188660	P(LINC00319|lncRNA-Topic 8) = 0.02	[44]
**lncRNA-Topic 13**		
**lncRNA_ID**	**Probability of association to topic**	**Ref.**
MALAT1 = ENSG00000251562	P(MALAT1|lncRNA-Topic 13) = 0.60	[36]
NEAT1 = ENSG00000245532	P(NEAT1|lncRNA-Topic 13) = 0.40	[37]
**lncRNA-Topic 15**		
**lncRNA_ID**	**Probability of association to topic**	**Ref.**
MIR205HG = ENSG00000230937	P(MIR205HG|lncRNA-Topic 15) = 0.66	[45,46]
**lncRNA-Topic 19**		
**lncRNA_ID**	**Probability of association to topic**	**Ref.**
TP53TG1 = LINC00096 = ENSG00000182165	P(TP53TG1|lncRNA-Topic 19) = 0.15	[47,48,49]
OSER1-DT = OSER1-AS1 = ENSG00000223891	P(OSER1-DT|lncRNA-Topic 19) = 0.03	[47]

**Table 3:** AMI scores computed to evaluate the concordance between the cell clusters derived from integrated clustering and single-omics clustering.

Experiment 1	Experiment 2	AMI
Seurat WNN	Seurat mRNA	0.855
Seurat WNN	Seurat lncRNA	0.569
multibranch SBM mRNA-lncRNA	hsBM-mRNA	0.492
multibranch SBM mRNA-lncRNA	hSBM-lncRNA	0.431

## Data Availability

The original data presented in the study are openly available in the NCBI GEO repository GSE117309; scripts to reproduce all the analyses and figures presented in this paper are provided in the gitHub repository https://github.com/sysbio-curie/topic_modeling_lncRNAs (accessed on 1 March 2024).

## Supplementary Materials

The following supporting information can be downloaded at: https://www.mdpi.com/article/10.3390/cancers16071350/s1, Figure S1: Coefficient of variation of the mRNAs and lncRNAs; Figure S2: Minimum description length reached with varying numbers of initializations; Figure S3: Distribution of the length of the gene sets in the MSigDB database and lncSEA database; Figure S4: Hierarchical clustering of cells from seven healthy donors of breast tissue.
