# Supplementary material for: Identification of Interpretable Clusters and Associated Signatures in Breast Cancer Single-Cell Data: A Topic Modeling Approach
Source: Cancers (Basel). 2024 Mar 29;16(7):1350. doi: 10.3390/cancers16071350 (PMC11011054; doi:10.3390/cancers16071350)
Supplement: Supplementary file 1 [file cancers-16-01350-s001.zip › Supplementary Figure S1 .pdf]

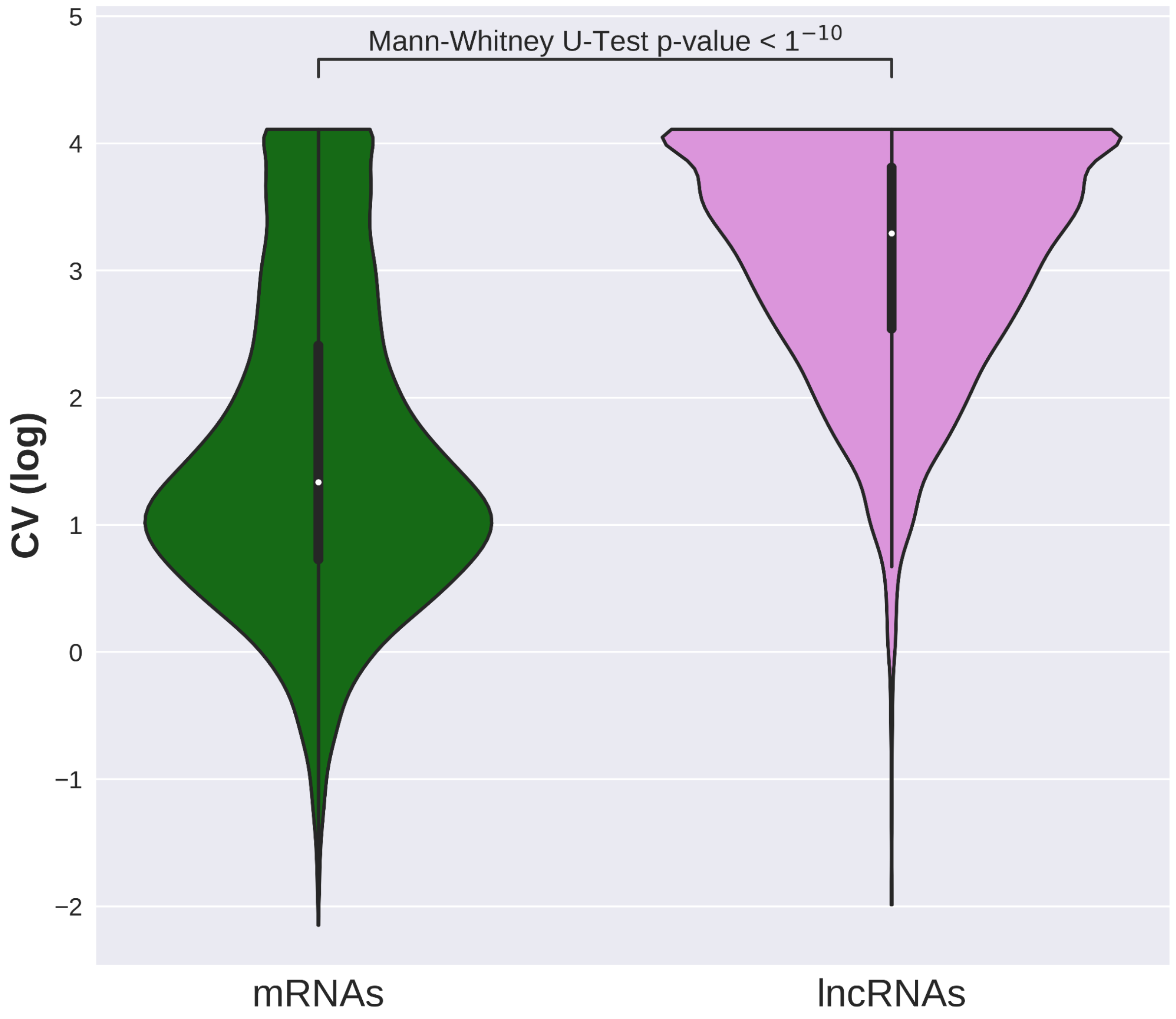

Supplementary Figure S1 Violin plots showing the distribution of the coefficient of variation of the mRNAs (green) and lncRNAs (pink). The p-value indicates the outcome of the Mann-Whitney U-Test.
