# Supplementary material for: Identification of Interpretable Clusters and Associated Signatures in Breast Cancer Single-Cell Data: A Topic Modeling Approach
Source: Cancers (Basel). 2024 Mar 29;16(7):1350. doi: 10.3390/cancers16071350 (PMC11011054; doi:10.3390/cancers16071350)
Supplement: Supplementary file 1 [file cancers-16-01350-s001.zip › Supplementary Figure S2.pdf]

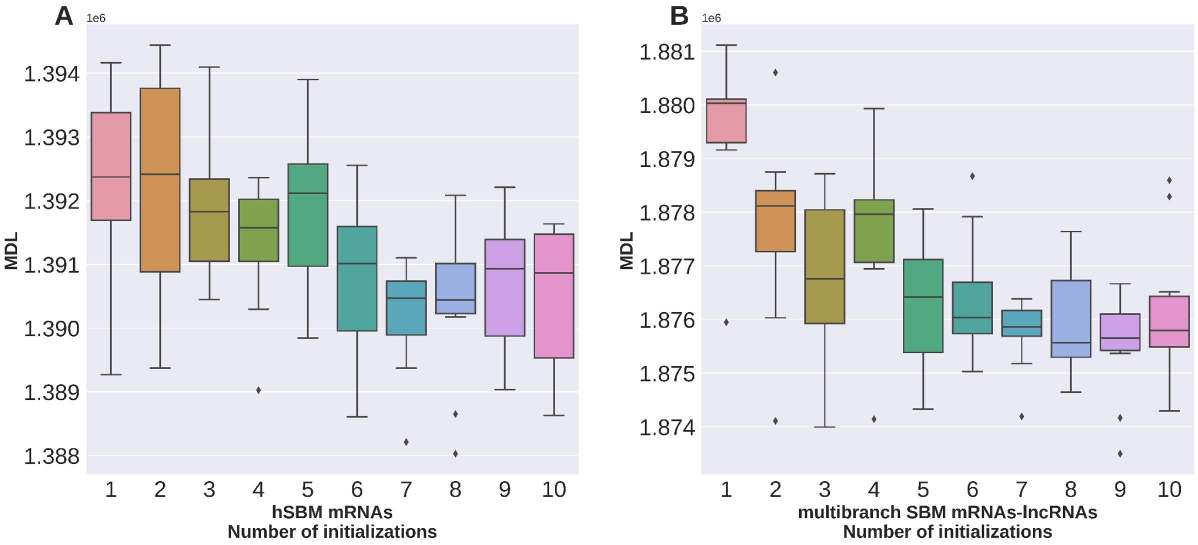

Supplementary Figure S2 Boxplot of the minimum description length (MDL) reached varying the numbers of initializations for the experiment hSBM-mRNA (A) and the multibranch experiment (B). Both hSBM and multibranch SBM show an elbow at seven initializations.
