# Supplementary material for: Identification of Interpretable Clusters and Associated Signatures in Breast Cancer Single-Cell Data: A Topic Modeling Approach
Source: Cancers (Basel). 2024 Mar 29;16(7):1350. doi: 10.3390/cancers16071350 (PMC11011054; doi:10.3390/cancers16071350)
Supplement: Supplementary file 1 [file cancers-16-01350-s001.zip › Supplementary Figure S3.pdf]

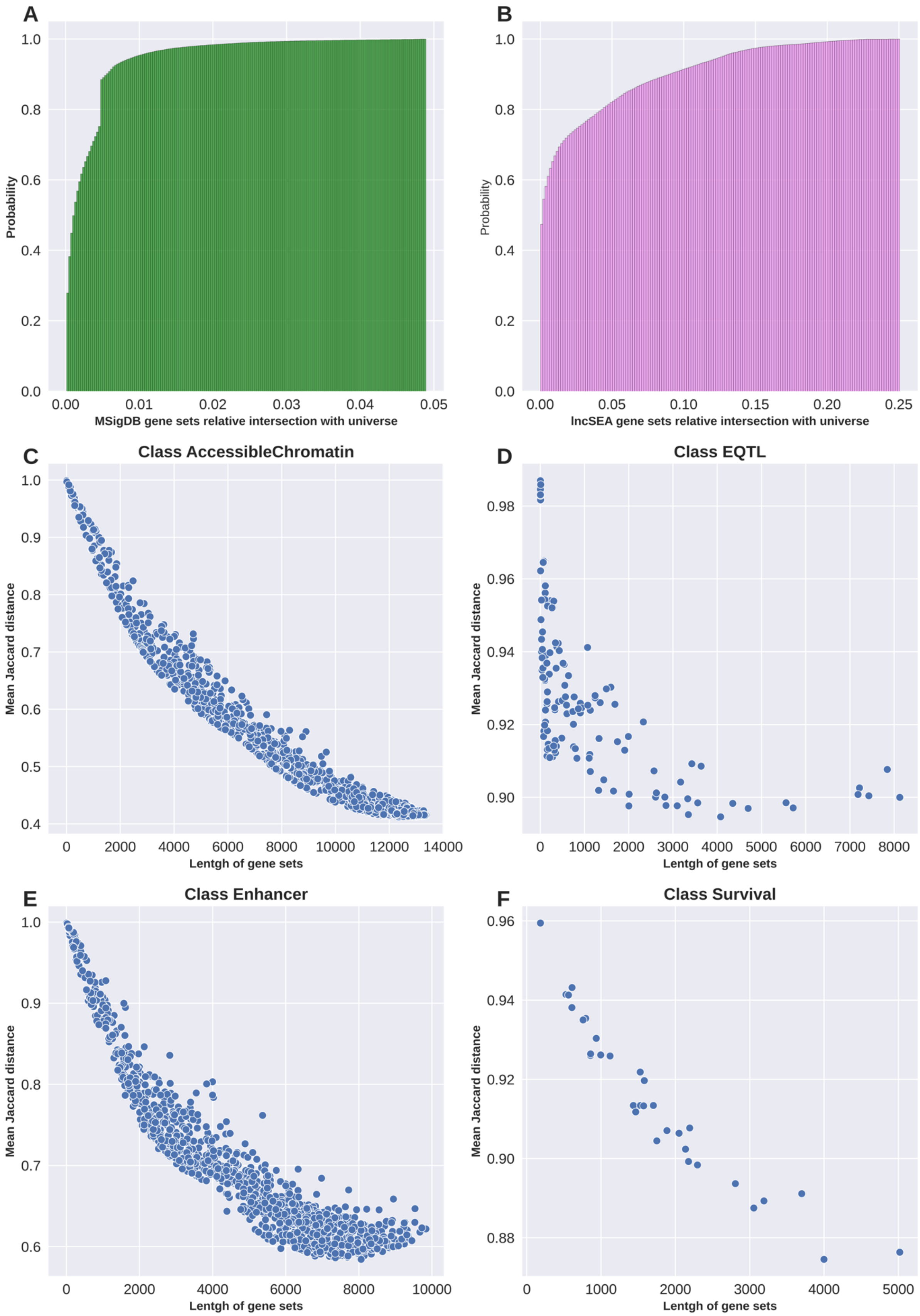
A
Probability
MSigDB gene sets relative intersection with universe
B
Probability
lncSEA gene sets relative intersection with universe
C
Class AccessibleChromatin
Mean Jaccard distance
Lentgh of gene sets
D
Class EQTL
Mean Jaccard distance
Lentgh of gene sets
E
Class Enhancer
Mean Jaccard distance
Lentgh of gene sets
F
Class Survival
Mean Jaccard distance
Lentgh of gene sets

Supplementary Figure S3 Distribution of the length of the gene sets in the MSigDB database for the mRNAs (A) and lncSEA database for lncRNAs (B). The latter consists of very longer gene sets in comparison to the former. Four examples of the relationship between the length of the gene sets and the mean similarity, measured with the Jaccard distance, among the sets (C, D, E, F). The decreasing monotonic relationship allows for the using of the filter based on the length instead of the Jaccard distance since the latter is highly computationally demanding.
