# Supplementary material for: Identification of Interpretable Clusters and Associated Signatures in Breast Cancer Single-Cell Data: A Topic Modeling Approach
Source: Cancers (Basel). 2024 Mar 29;16(7):1350. doi: 10.3390/cancers16071350 (PMC11011054; doi:10.3390/cancers16071350)
Supplement: Supplementary file 1 [file cancers-16-01350-s001.zip › Supplementary Figure S4.pdf]

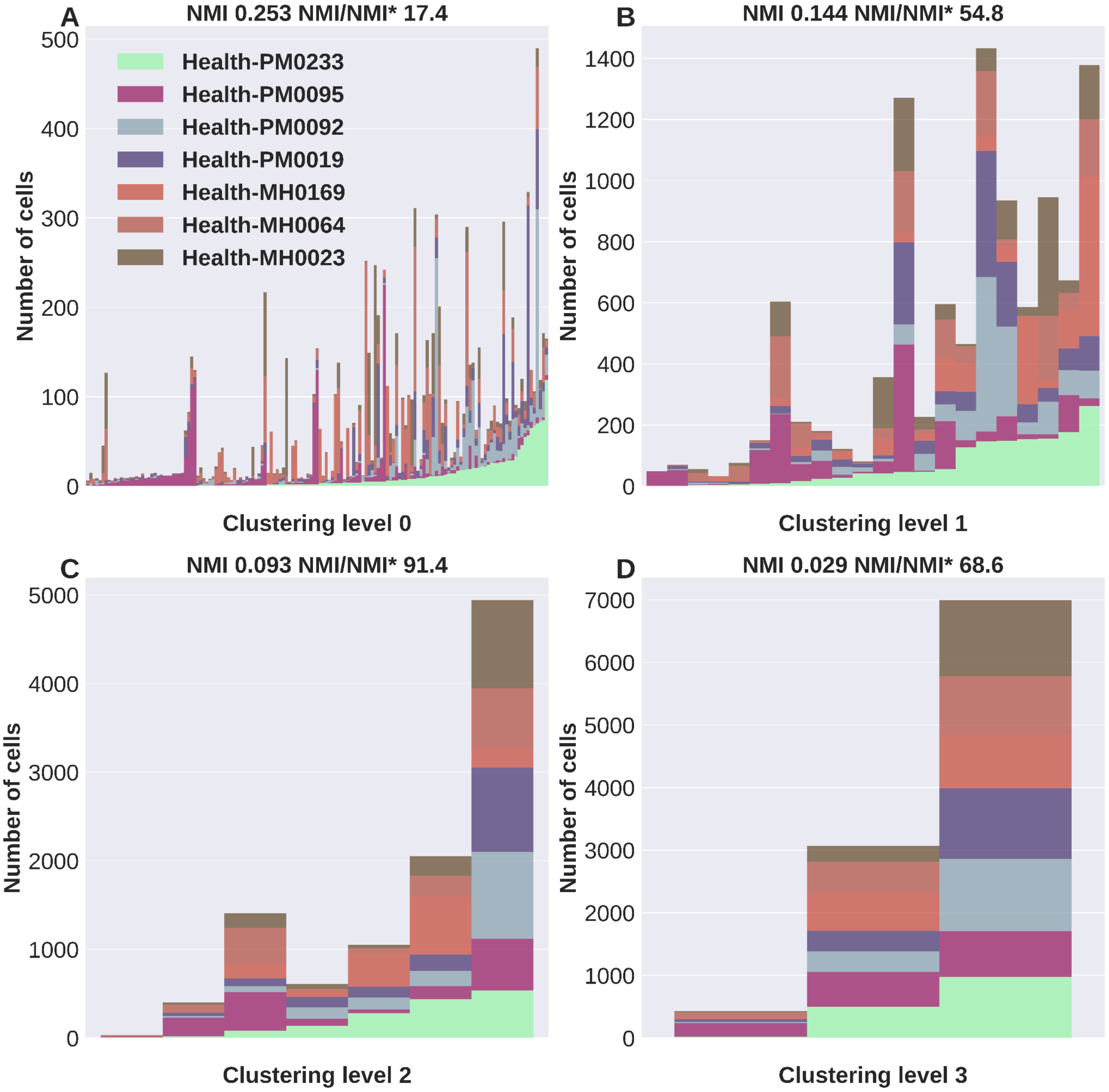

Supplementary Figure S4 Hierarchical clustering of cells from seven healthy donors of breast tissue. Each panel shows the clusters belonging to one level of the hierarchy. We applied the multibranch algorithm creating the three usual partitions: cells, mRNAs and lncRNA. The low values of NMI and NMI/NMI* and the homogeneous composition of each cluster prove that the algorithm cannot recognize the donor.
